# *VEGFA* Haplotype and VEGF-A and VEGF-R2 Protein Associations with Exudative Age-Related Macular Degeneration

**DOI:** 10.3390/cells11060996

**Published:** 2022-03-15

**Authors:** Alvita Vilkeviciute, Dzastina Cebatoriene, Loresa Kriauciuniene, Rasa Liutkeviciene

**Affiliations:** 1 Neuroscience Institute, Lithuanian University of Health Sciences, Medical Academy, Eiveniu St. 2, LT-50161 Kaunas, Lithuania; loresa.kriauciuniene@lsmuni.lt (L.K.); rasa.liutkeviciene@lsmuni.lt (R.L.); 2 Medical Academy, Lithuanian University of Health Sciences, A. Mickeviciaus St. 9, LT-44307 Kaunas, Lithuania; dzastina.cebatoriene@lsmu.lt

**Keywords:** age-related macular degeneration, *VEGFA*, haplotype, serum concentration, rs1570360, rs699947, rs3025033, rs2146323

## Abstract

Our study aimed to reveal the associations between *VEGFA* SNPs (rs1570360, rs699947, rs3025033, and rs2146323), their haplotypes, VEGF-A and VEGF-R2 serum concentrations, and early and exudative AMD. A total of 339 subjects with early AMD and 419 with exudative AMD groups, and 374 healthy subjects, were genotyped for four *VEGFA* SNPs (rs1570360, rs699947, rs3025033, and rs2146323). VEGF-A and VEGFR-2 serum concentrations were measured in exudative AMD and controls. The results revealed that rs3025033 G allele was significantly associated with lower odds of exudative AMD under the dominant model (OR = 0.67; 95% CI: 0.49–0.80; *p* = 0.0088) and additive (OR = 0.7; 95% CI: 0.54–0.90; *p* = 0.0058) models after Bonferroni correction. In the female group, rs3025033 AG genotype was associated with exudative AMD under the codominant model (OR = 0.57; 95% CI: 0.37–0.87; *p* = 0.009) and G allele under the dominant (OR = 0.55; 95% CI: 0.37–0.82; *p* = 0.0032) and additive models (OR = 0.60; 95% CI: 0.42–0.84; *p* = 0.0028). Haplotype analysis revealed that individuals carrying rs1570360, rs699947, rs3025033, and rs2146323 haplotype A-A-G-A had decreased risk of exudative AMD (OR = 0.46, 95% CI: 0.23–0.90; *p* = 0.023). The VEGF-A and VEGF-R2 serum concentrations did not differ between study groups; we found that patients with exudative AMD carrying at least one C allele at rs699947 have statistically significantly higher VEGF-A serum concentrations compared to AA genotype carriers (485.95 (945.93) vs. 194.97 (-), respectively, *p* = 0.046). In conclusion, we found that *VEGFA* rs3025033 and haplotype rs1570360A-rs699947A-rs3025033G- rs2146323A play a protective role for exudative AMD in the Caucasian population. Furthermore, rs699947 is associated with elevated VEGF-A serum concentrations in exudative AMD.

## 1. Introduction

Age-related macular degeneration (AMD) is widely described as a multifactorial, progressive, neurodegenerative disease of the macula, causing loss of visual functions leading to blindness. The macula is a part of the retina where the photoreceptors, responsible for visual acuity and colour perception, are concentrated [1]. Most eye function impairments are associated with age-related alterations and, together with the other risk factors such as environmental and genetic factors, lead to severe eye conditions [2]. Exponential population ageing is a recent worldwide problem affecting human health, including eye diseases. It is known that AMD affects about 10% of people older than 65 years and more than 25% of people older than 75 years in developed countries [2]. The previous study’s projection shows that the number of people with early AMD will increase up to 21.5 million and late AMD up to 4.8 million in Europe [3], while worldwide the numbers are expected to increase dramatically, even up to 288 million in 2040 [3].

AMD can be classified based on histopathological changes [4]. Early and intermediate AMD often may not cause any symptoms. Early AMD signs include small drusen (protein and lipid deposit) formation between the retinal pigment epithelium (RPE) and Bruch’s membrane [2]. The intermediate form is described as a presence of at least one large drusen, numerous medium-sized drusen, or geographic atrophy (GA) without extension to the center of the macula with mild symptoms, such as mild blurriness, in their central vision or trouble seeing in low lighting. The advanced AMD is divided into dry or atrophic AMD with the GA of the RPE, and neovascular or exudative AMD is diagnosed when choroidal neovascularization with detachments in the RPE haemorrhages and/or scars appear and cause progressive blurring or other central vision impairments [5].

Considering the multifactorial AMD pathogenesis, drusogenesis and oxidative stress remain the essential processes [6]. Accumulation of molecular debris, including lipid and protein particles as well as reactive oxygen species (ROS) leading to chronic inflammation, cause the irreversible damage of RPE cells and photoreceptors in the macula [7,8], and also the breakdown of the blood–ocular barrier, which is a normal function to protect the eye [9].

The above-mentioned pathological alterations cause ocular damage, resulting in cytokine release and pro-angiogenic and anti-angiogenic factor disbalance, including increased vascular endothelial growth factor A (VEGFA) production, which plays a crucial role in angiogenesis and vascular permeability. Moreover, vascular leakage and inflammation caused by excessive VEGFA release were found to play a critical role for choroidal neovascularisation and exudative AMD development [7,10].

VEGFA can regulate angiogenesis in the vascular endothelium binding two types of VEGF family receptors: VEGF-R1, encoded by FMS-related tyrosine kinase 1; *FLT1* gene (OMIM* 165070) and VEGF-R2, encoded by kinase insert domain receptor; *KDR* gene (OMIM* 191306) [11]. One of these high-affinity receptor tyrosine kinases, VEGF-R2, is a primary angiogenic receptor associated with VEGFA-stimulated vascular permeability [12,13], and the VEGF-R1 focuses on endogenous VEGFA inhibition [12,14].

While only the exudative AMD is already treated with anti-VEGFA inhibitors, in recent years, the VEGFA and its signaling pathway have been targeted for the most effective therapy development [15], including the other molecules based on the pathogenic processes in which they are involved [16].

Although AMD involves many risk factors, a substantial genetic contribution reveals the genetic marker associations with AMD susceptibility [17], including a relationship between the *VEGFA* and *VEGFR2* gene variants and AMD as well [18,19,20,21]. Regarding the significant associations and conflicting results between genetic markers and AMD [22] in previous studies, we aimed to investigate the associations between four single nucleotide polymorphisms (SNPs) in *VEGFA* (OMIM* 192240), and VEGF-A and VEGFR-2/KDR protein roles in AMD in the Caucasian population to focus on a potential tool for early diagnosis.

## 2. Materials and Methods

### 2.1. Study Groups

This study was conducted after the approval of the Ethics Committee for Biomedical Research, Lithuanian University of Health Sciences (No. BE-2-/48; 8 October 2018).

Study groups consisted of subjects admitted to the Hospital of Lithuanian University of Health Sciences Ophthalmology Department for ophthalmological evaluation. In our study, 1132 participants were enrolled: 339 subjects in early AMD and 419 in the exudative AMD group. The Control group was formed of 374 healthy subjects (Table 1). Using the global AMD prevalence (8.7%) [23] and the minor allele frequencies from [24], we calculated that our collected sample sizes for the early, exudative AMD and control groups were sufficient to reach 80% or higher statistical power for the selected *VEGFA* SNP analysis.

An ophthalmological evaluation was performed for all the study subjects, and data about general health and other diseases were obtained from study subjects. All study subjects agreed to participate and signed an informed consent form.

### 2.2. AMD Groups

All the AMD patients underwent optical coherence tomography (OCT), and optical coherence tomography angiography (OCT-A) was performed in patients suspected of having late AMD after the OCT examination. Exudative AMD was diagnosed in one eye of the study subjects.

Age-Related Eye Disease Study (AREDS) classification was used for AMD diagnosis and has been described previously [25].

Early AMD was defined as a combination of multiple small drusen and several intermediate drusen (63–124 μm in diameter) or retinal pigment epithelial abnormalities.

Intermediate AMD was characterized by the presence of extensive intermediate drusen and at least one large (giant) druse (≥125 μm in diameter) or geographic atrophy (GA) not involving the center of the fovea.

Advanced AMD was characterized by GA involving the fovea and/or any of the features of neovascular AMD.

### 2.3. The Control Group

Subjects without ophthalmologic pathologies and patients after senile cataract surgeries (without any other ocular comorbidities) were included in the control group. The following exclusion criteria were also described in previous publications [26]:

(1) unrelated eye disorders, e.g., high refractive error, cloudy cornea, lens opacity (nuclear, cortical, or posterior subcapsular cataract) except minor opacities, keratitis, acute or chronic uveitis, glaucoma, or diseases of the optic nerve;
(2) systemic illnesses, e.g., diabetes mellitus, malignant tumors, systemic connective tissue disorders, chronic infectious diseases, hypertension, coronary artery disease, stroke or conditions following organ or tissue transplantation;
(3) ungraded colour fundus photographs resulting from obscuring the ocular optic system or because of fundus photograph quality.

### 2.4. DNA Extraction

DNA extraction was carried out at the Laboratory of Ophthalmology, Neuroscience Institute, LUHS. The DNA was extracted from 200 µL peripheral venous blood samples utilizing silica-based membrane technology, using a genomic DNA extraction kit (GeneJET Genomic DNA Purification Kit, Thermo Fisher Scientific, Vilnius, Lithuania), based on the manufacturer’s recommendations.

### 2.5. SNP Selection

**Figure 1:**
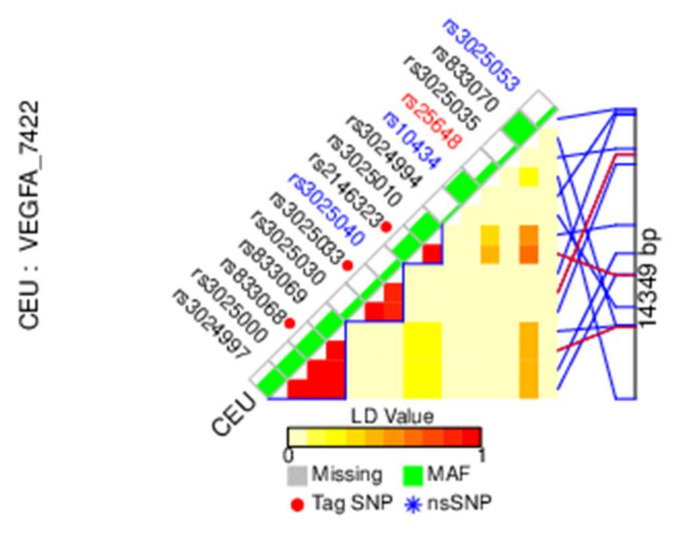
Haploblocks in CEU population, HapMap data. r^2^ = 0.8 set; a minimum number of SNPs tagged by each tag SNP = 2.

In the present study, two tag SNPs (intronic variants rs3025033A/G and rs2146323A/C) covering two haploblocks (Figure 1) (rs833068 have already been analyzed in our previous research study) [26] were selected from the CEU population using the public HapMap database. The pairwise option of the online Tag SNP tool was used with the following settings: r^2^ = 0.8 set and the minimum number of SNPs tagged by each tag SNP was 2.

Two other *VEGFA* promoter polymorphisms, −2578C/A (rs699947) and −1154G/A (rs1570360), were selected additionally based on the previous inconsistent results [27] and potential multiple SNP associations with the AMD [19].

Finally, 4 SNPs in *VEGFA* gene were selected for genotyping and further analysis: rs1570360 (chromosome6:43770093 (GRCh38)), rs699947 (chromosome6:43768652 (GRCh38)), rs3025033 (chromosome6:43783338 (GRCh38)), rs2146323 (chromosome6:43777358 (GRCh38)).

### 2.6. Genotyping

The genotyping of four *VEGFA* gene polymorphisms, rs1570360, rs699947, rs3025033, and rs2146323, was carried out at the Laboratory of Ophthalmology, Neuroscience Institute, LUHS. The identification of all single-nucleotide polymorphisms was performed on a “StepOnePlus” real-time PCR quantification system (Thermo Fisher Scientific, Singapore) using predesigned TaqMan^®^ Genotyping assays (Thermo Fisher Scientific, Pleasanton, CA, USA) according to the manufacturer‘s recommendations. Genotyping results were obtained using Genotyping program on the StepOne software.

### 2.7. Quality Control of Genotyping

A total of 5% randomly chosen samples were repetitively genotyped for all four SNPs to confirm the same rate of genotypes from initial and repetitive genotyping.

### 2.8. Serum VEGF-A and VEGF-R2 Concentration Measurement

Obtained serum was partitioned into 200 μL aliquots to Eppendorf tubes and frozen at −80 °C. Serum VEGF-A and VEGF-R2 concentrations were determined by sandwich enzyme-linked immunosorbent assays, using the Human VEGF-A ELISA (Cat. No. BMS277-2) and VEGF-R2/KDR ELISA (Cat. No. BMS2019) kits following the manufacturer’s instructions. Results were observed using a Multiskan FC microplate photometer (Thermo Scientific, Waltham, MA, USA) at 450 nm. VEGF-A assay sensitivity was 7.9 pg/mL, VEGF-R2/KDR—7 pg/mL.

### 2.9. Statistical Analysis

Statistical analysis was performed using the SPSS/W 27.0 software (Statistical Package for the Social Sciences for Windows, Inc., Chicago, IL, USA). Continuous data (age, protein serum level data distributions were evaluated for normality by the Kolmogorov–Smirnov test. Continuous variables presented as median with interquartile range (IQR) based on data distribution. The Mann–Whitney test was used to compare two groups for non-normally distributed data.

Categorical data (gender, genotype, and allele distributions) are presented as absolute numbers with percentages in brackets and compared between the early, exudative, AMD, and control groups using the *chi*-*square* (χ^2^) test. Hardy–Weinberg equilibrium was evaluated to compare the observed and expected frequencies of *VEGFA* rs1570360, rs699947, rs3025033, and rs2146323 using χ^2^ test in the control group as well.

The impact of SNPs on early and exudative AMD was evaluated using binomial logistic regression analysis. Results are presented as odds ratios (OR) with 95% confidence interval (CI) and adjusted by covariate effect for age in the exudative AMD groups. Logistic regression analysis results were expressed as genetic models (codominant: heterozygotes vs. major allele homozygotes and minor allele homozygotes vs. major allele homozygotes; dominant: minor allele homozygotes and heterozygotes vs. major allele homozygotes; recessive: minor allele homozygotes vs. major allele homozygotes and heterozygotes; overdominant: heterozygotes vs. major allele homozygotes and minor allele homozygotes); the additive model was used to evaluate the impact of each minor allele on AMD: major allele homozygotes vs. heterozygotes *vs.* minor allele homozygotes. The best genetic model selection was based on the Akaike information criterion (AIC); therefore, the best genetic models had the lowest AIC values. We introduced an adjusted significance threshold for multiple comparisons alpha = 0.0125 (0.05/4, since we analyzed four SNPs in the *VEGF-A* gene) [28]. 

Haplotype analysis was performed in the early AMD and control groups, and exudative AMD and control groups separately, using online SNPStats software (https://www.snpstats.net/snpstats/ (accessed on 15 December 2021)) [29]. Linkage disequilibrium (LD) analysis was assessed by D’ and r^2^ measures. The associations between the haplotypes and different AMD forms were calculated by logistic regression and presented as ORs and 95% CI and values adjusted for age in exudative AMD analysis. Haplotypes with less than 1% frequencies were pooled into one group and described as “rare”. A two-sided test with a value less than 0.05 was considered statistically significant. Graphs were performed using GraphPad Prism version 9.0.0 for Mac, GraphPad Software, San Diego, CA, USA, www.graphpad.com (accessed on 21 February 2022).

## 3. Results

### 3.1. VEGFA (rs1570360, rs699947, rs3025033, and rs2146323) Genotype and Allele Associations with Early and Exudative AMD

*VEGFA* SNPs’ genotype distributions were evaluated in the control group using Hardy–Weinberg equilibrium (HWE). Three SNPs were in HWE (*p* > 0.05), but rs1570360 did not conform the HWE requirements (*p* < 0.001) (Table 2).

Analysis of genotype and allele distributions showed statistically significant differences of rs3025033 genotypes (AA, AG, and GG) between exudative AMD and control groups (69.2%, 27.7% and 3.1% vs. 61%, 33.4%, and 5.6%, respectively, *p* = 0.029) (Table 2). Still, these results did not survive Bonferroni correction.

Allele frequency analysis showed that G allele at rs3025033 was statistically significantly less frequent in the exudative AMD group than controls (16.9% vs. 22.3%, respectively, *p* = 0.007) (Table 2).

Binomial logistic regression revealed that G allele at rs3025033 was significantly associated with lower odds of exudative AMD under the dominant model (OR = 0.67; 95% CI: 0.49–0.80; *p* = 0.0088) and additive (OR = 0.7; 95% CI: 0.54–0.90; *p* = 0.0058) model as best according to AIC value, even after Bonferroni correction (Table 3).

### 3.2. VEGFA (rs1570360, rs699947, rs3025033, and rs2146323) Genotype and Allele Associations with Early and Exudative AMD by Gender

We analyzed *VEGFA* (rs1570360, rs699947, rs3025033, and rs2146323) genotype and allele associations with early and exudative AMD by gender and found that rs3025033 association with exudative AMD remained only in females (Supplementary Material, Tables S1 and S2), but not in males (Supplementary Material S1, Tables S3 and S4). After the strict Bonferroni correction, three significant results remained. We found association between rs3025033 AG genotype and exudative AMD under the codominant model (OR = 0.57; 95% CI: 0.37–0.87; *p* = 0.009); we also revealed that G allele is associated with lower odds of exudative AMD for females under the dominant (OR = 0.55; 95% CI: 0.37–0.82; *p* = 0.0032) and additive models (OR = 0.60; 95% CI: 0.42–0.84; *p* = 0.0028) (Supplementary Material, Table S2). AIC value shows that the additive model was best for revealing rs3025033 association with exudative AMD in females (Supplementary Material, Table S2).

While the logistic regression analysis showed significant associations between rs2146323 and exudative AMD in females, the results did not survive Bonferroni correction (Supplementary Material, Table S2). No associations were found between rs2146323 and early AMD in females (Supplementary Material S1, Tables S1 and S2) nor males (Supplementary Material, Tables S3 and S4).

We also compared the genotype and allele frequencies between females with early AMD and exudative AMD and males with early AMD and exudative AMD. We found that allele G at rs3025033 was less frequent in females with exudative AMD than in females with early AMD (14.4% vs. 20%, respectively *p* = 0.019). Still, the result did not survive Bonferroni correction (Supplementary Material, Table S1).

### 3.3. Haplotype Analysis

Haplotype analysis was performed in separate groups of AMD. Pairwise linkage disequilibrium (LD) between studied polymorphisms was observed (Table 4).

Haplotype analysis revealed that there is no association between *VEGFA* haplotypes and early AMD (Table 5), but individuals carrying rs1570360, rs699947, rs3025033, and rs2146323 haplotype A-A-G-A had decreased risks of exudative AMD (OR = 0.46, 95% CI: 0.23–0.90; *p* = 0.023) (Table 6).

### 3.4. VEGF-A and VEGF-R2/KDR Serum Concentration Analysis

Serum protein concentrations were measured in 20 patients with exudative AMD before treatment and 21 control group samples. The Control group for VEGF-A and VEGF-R2/KDR serum concentration measurement consisted of subjects considering the age and gender distributions based on the exudative AMD group.

**Figure 2:**
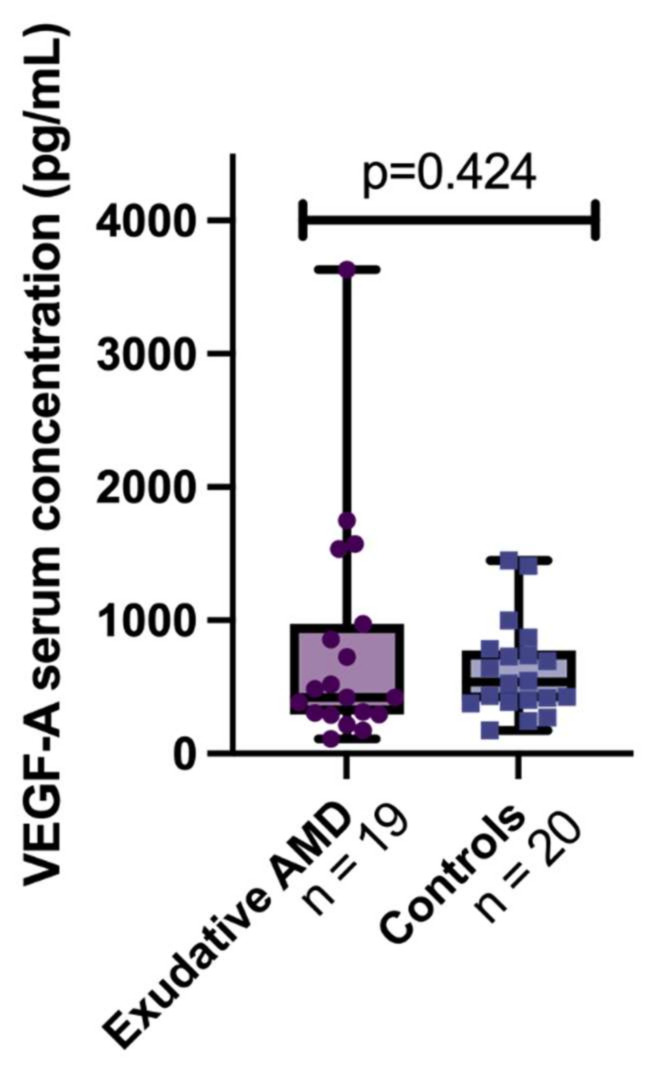
VEGF-A serum concentrations between groups. The bars represent the median with interquartile range (first quartile and third quartile) and whiskers from min to maximum values. VEGF-A serum concentration in exudative AMD group: 422.674 (677.02) pg/mL and control group: 615.489 (425.49) pg/mL. Mann–Whitney-U test, *p* = 0.424.

We compared the VEGF-A serum concentrations between exudative AMD and control groups but did not find a significant difference (422.674 (677.02) vs. 615.489 (425.49), respectively, *p* = 0.424) (Figure 2).

**Figure 3:**
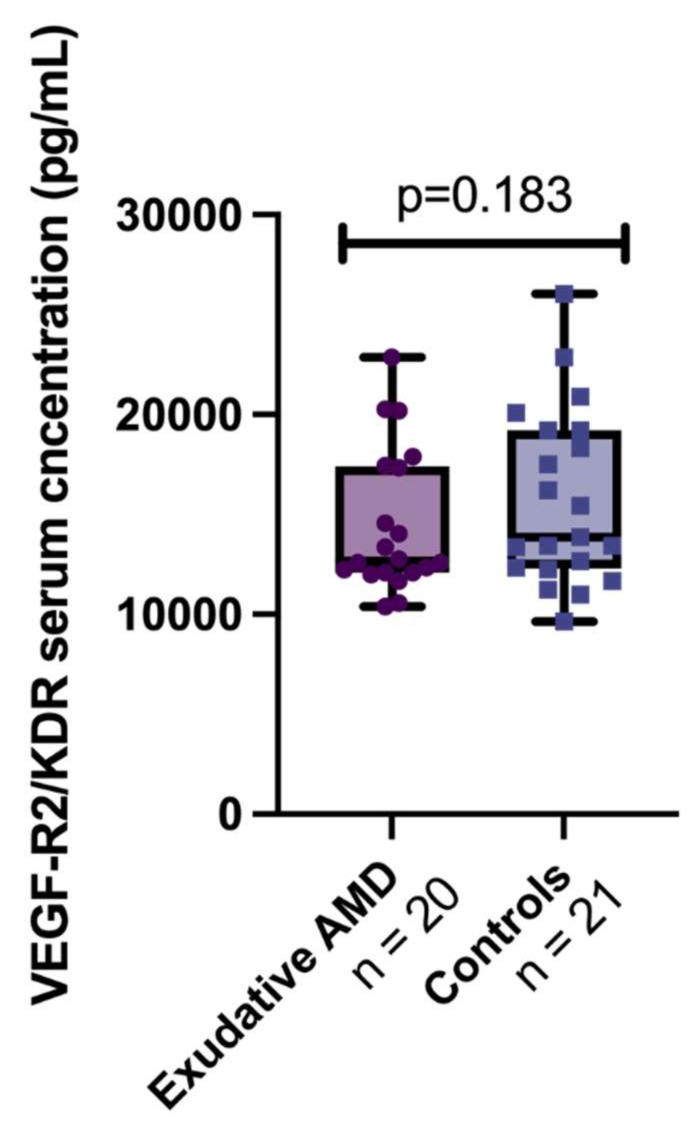
VEGF-R2/KDR serum concentrations between groups. The bars represent the median with interquartile range (first quartile and third quartile) and whiskers from minimum to maximum. VEGF-R2/KDR serum concentration in exudative AMD group: (12,759.2 (5358.85) pg/mL and control group: 15,428.35 (6698.03) pg/mL. Mann–Whitney-U test, *p* = 0.183.

We also compared the VEGF-R2/KDR serum concentrations between exudative AMD and control groups, but there was also no statistical difference (12,759.2 (5358.85) vs. 15,428.35 (6698.03), respectively, *p* = 0.183) (Figure 3).

### 3.5. VEGF-A and VEGF-R2/KDR Concentrations by VEGFA Genotypes

We also performed VEGF-A and VEGF-R2/KDR serum concentration and *VEGFA* rs1570360, rs699947, rs302503, and rs2146323 genotype association analysis and found that patients with exudative AMD carrying at least one C allele at rs699947 have statistically significantly higher VEGF-A serum concentrations compared to wild-type allele A homozygous genotypes carriers (485.95 (945.93) vs. 194.97 (-), respectively, *p* = 0.046) (Table 7). However, there were only two subjects with the AA genotype.

## 4. Discussion

This study analyzed four SNPs in the *VEGFA* gene and their associations with early and exudative AMD. Study results revealed that the G allele at rs3025033 was significantly associated with lower odds of exudative AMD. We also found that these associations with exudative AMD remained only in females but not in males, suggesting the potential gender role in AMD development. Furthermore, the differences of rs3025033 allele G frequencies were observed between females with exudative AMD and females with early AMD, but the results did not survive Bonferroni correction. Moreover, associations between rs2146323 and exudative AMD in females were found, but the results did also not survive Bonferroni correction.

A haplotype of *VEGFA* SNPs analysis revealed that individuals carrying rs1570360, rs699947, rs3025033, and rs2146323 haplotype A-A-G-A had decreased risk of exudative AMD, showing a protective role of this haplotype. No studies have previously included all these SNPs in AMD analysis. Only Mori et al. (2010) analyzed *VEGFA* −116A, rs1570360 associations with AMD, but did not reveal significant results [27]. Another three studies examined rs1570360 associations with exudative AMD treatment efficacy but not with the AMD occurrence [30,31,32].

*VEGFA* promoter polymorphism −2578C/A (rs699947) is widely studied in a large number of angiogenesis-associated diseases, including many different types of cancers [33,34,35] and their response to anti-VEGFA agent treatment [36].

This SNP was also studied in patients with AMD, but statistically significant differences in the genotypic distribution of *VEGFA* rs699947 were not found, and any significant associations were revealed [20,27,37,38]. Scientists also tried to find the differences between the AMD subtypes comparing neovascular and atrophic AMD groups but did not reveal significant results [39].

Previous results were found in much smaller study groups. Still, our study confirmed those findings in bigger samples groups, including 339 subjects in early AMD and 419 in the exudative AMD groups, and 374 subjects in the control group.

Other research studies have analyzed rs699947 associations with anti-VEGFA treatment [30,31,40,41,42] or photodynamic therapy response [32,43,44] but conflicting results suggest that other risk factors [45] or SNP combinations may be associated with the AMD treatment as well [46,47]. Our analysis of SNPs and VEGF-A and VEGF-R2 serum concentrations revealed that carriers of at least one C allele at rs699947 have statistically significantly higher VEGF-A serum concentrations compared to wild-type allele A homozygous genotype carriers (485.95 (945.93) vs. 194.97 (-), respectively, *p* = 0.046).

Bulgu et al. (2014) included in their study an intronic VEGFA variant rs3025033 and genotyped it for 82 AMD patients and 80 controls. Unfortunately, about 98% of genotypes were determined as AA, so no further statistical analysis was performed [48].

Li et al. (2021) analyzed the rs3025033 effect on VEGF165b protein production but did not reveal significant results; on the other hand, they showed that this SNP promoted cell proliferation in human retinal vascular endothelial cells (hRVECs) [49].

Another study performed by Immonen et al. (2010) analyzed the associations between rs3025033 and photodynamic therapy response, but no associations were revealed [44].

In contrast, the frequencies of the *VEGFA* +5092, rs2146323 were significantly different in photodynamic therapy non-responders and responders [44]. However, similar frequencies of this SNP were found between the exudative AMD patients and controls [37] and any AMD patients and controls [48]. Furthermore, it was shown that rs2146323 C allele was protective against dry AMD, and the allele A was associated with the disease. Moreover, there was a significant difference in the genotype frequencies of this SNP between the wet type of AMD and dry type AMD [48]. Our study results showed associations between rs2146323 and exudative AMD only in females, but these results did not survive when we applied strict Bonferroni correction. Furthermore, we did not find any associations between this SNP and VEGF-A or VEGF-R2 serum concentrations.

Other widely studied SNPs show significant associations with the AMD and highlight the possible *VEGFA* rs1413711 and rs833061 polymorphisms contributions to AMD susceptibility [50].

Bulgu et al. (2014) found that the presence of ancestral allele (G) in rs1413711 was protective for all AMD patients, and the AA genotype was a risk factor for AMD and even a highly increased risk factor for dry AMD [48]. Opposite results were shown in another study: SNP +674, rs1413711 CC genotype was significantly associated with a higher risk of exudative AMD [19]. The conflicting results of these studies may be explained by differences in the size of study groups and populations (Turkish and Caucasian of Northern European origin).

Habibi et al. (2014) showed that *VEGFA* +405, rs2010963 CC and *VEGFA* +936, rs3025039 TT genotype frequencies were higher in Tunisian AMD patients than in controls [51].

These results confirmed the previous associations between *VEGFA* +936, rs3025039, and wet AMD in the Japanese population [52]. Deeper analysis showed that genotype TT for rs3025039 was associated with elevated VEGF-A protein serum levels [20].

Moreover, an extensive analysis of *VEGFA* promoter and gene polymorphisms showed that SNPs +674, +4618, +5092, +9162, and +9512 their haplotypes, CTCCT and TCACC, were associated with a 15-fold increased risk of exudative AMD and the promoter SNPs −460T, −417T, −172C, −165C, −160C, −152G, −141A, −116A, +405C haplotype was associated with about an 18-fold greater risk [19]. Two of these polymorphisms were included in our study and, together with another two SNPs, revealed a protective haplotype for exudative AMD. It is important to elucidate the SNP combinations and their role in AMD to understand the pathogenesis and possible treatment strategies better.

VEGF-A serum levels, as well as VEGFA SNPs, are widely studied in AMD patients. While we did not find any statistical differences in VEGF-A serum levels between the exudative AMD patients and controls, we confirmed results from several other studies which included total AMD patients or only exudative AMD patients, consisting of 27 to 71 samples per group in different populations [53,54,55,56,57,58,59,60]. Further studies revealed significantly elevated VEGF-A levels in exudative AMD patients compared to controls [61,62]. Significantly elevated VEGF-A levels in total AMD patients compared to controls were also found in a few studies [20,51,63].

VEGF-R2 protein associations are not so widely analyzed as VEGF-A, but conflicting results were also found. Örnek et al. (2016) found that decreased VEGFR-2 serum levels were associated with both dry and wet type AMD. Furthermore, negative correlations between VEGFR-2 with foveal retinal thickness in AMD patients and a significant positive correlation with subfoveal choroidal thickness revealed the possible VEGF-R2 role in AMD development [64]. Another two studies showed opposite associations and found elevated VEGFR2 protein levels in patients with exudative AMD group than in the controls [65,66], suggesting the role of VEGFR-2 in the pathogenesis of AMD.

## 5. Conclusions

In conclusion, we found that rs3025033 polymorphism of *VEGFA* and the haplotype rs1570360A-rs699947A-rs3025033G-rs2146323A can play a protective role for exudative AMD in our population. Furthermore, the C allele at rs699947 is associated with elevated VEGF-A serum concentrations in exudative AMD patients. Since the genetic variations were associated with exudative AMD, our findings need to be replicated in additional studies. While the main strength of our study was a large number of early and exudative AMD patients, further studies are still needed to investigate the pharmacologic role of these angiogenesis-related markers in AMD therapy.

## Figures and Tables

**Table 1:** Demographic characteristics of study groups.

Characteristic	Early AMD N = 339	Exudative AMD N = 419	Control Group N = 374	*p* Value
Gender Males, n (%) Females, n (%)	104 (30.7) 235 (69.3)	149 (35.6) 270 (64.4)	139 (37.2) 235 (62.8)	0.068 * 0.639 **
Age, median (IQR)	73 (13)	77 (10)	72 (10)	0.382 * <0.001 **

IQR—interquartile range; *p*—significance level when *p* = 0.05; * Early AMD vs. Control group; ** Exudative AMD vs. Control group.

**Table 2:** Frequencies of *VEGFA* (rs1570360, rs699947, rs3025033, and rs2146323) genotypes and alleles in early AMD, exudative AMD, and control groups.

SNP Genotype/Allele	Early AMD N = 339 n (%)	Exudative AMD N = 419 n (%)	Control Group N = 374 n (%)	HWE *p* Value	*p* Value *	*p* Value **	*p* Value ***
rs1570360				**<0.001**	0.186	0.136	0.512
GG	162 (47.8)	183 (43.7)	177 (47.3)				
AG	128 (37.8)	168 (40.1)	125 (33.4)				
AA	49 (14.5)	68 (16.2)	72 (19.3)				
G	452 (66.7)	534 (63.7)	479 (64)		0.298	0.897	0.234
A	226 (33.3)	304 (36.3)	269 (36)				
rs699947				0.168	0.317	0.589	0.183
AA	86 (25.4)	119 (28.4)	112 (29.9)				
AC	174 (51.3)	187 (44.6)	173 (46.30				
CC	79 (23.3)	113 (27)	89 (23.8)				
A	346 (51)	425 (50.7)	397 (53.1)		0.441	0.348	0.902
C	332 (49)	413 (49.3)	351 (46.9)				
rs3025033				0.482	0.399	**0.029**	0.242
AA	215 (63.4)	290 (69.2)	228 (61)				
AG	112 (33)	116 (27.7)	125 (33.4)				
GG	12 (3.5)	13 (3.1)	21 (5.6)				
A	542 (80)	696 (83.1)	581 (77.7)		0.296	**0.007**	0.119
G	136 (20)	142 (16.9)	167 (22.3)				
rs2146323				0.06	0.248	0.603	0.236
CC	142 (41.9)	191 (45.6)	158 (42.2)				
AC	157 (46.3)	169 (40.3)	157 (42)				
AA	40 (11.8)	59 (14.1)	59 (15.8)				
C	441 (65)	551 (65.8)	473 (63.2)		0.477	0.296	0.773
A	237 (35)	287 (34.2)	275 (36.8)				

HWE *p* value—Hardy–Weinberg equilibrium significance level *p* = 0.05; *p*—significance level and Bonferroni corrected significance level when *p* < 0.05/4; *p*-values marked with bold indicate statistically significant *p*-values. * Early AMD vs. Control group; ** Exudative AMD vs. Control group; *** Early AMD vs. Exudative AMD.

**Table 3:** Binomial logistic regression analysis of *VEGFA* (rs1570360, rs699947, rs3025033, and rs2146323) in early and exudative AMD and control groups.

	rs1570360						
	Early AMD vs. Control Group			Exudative AMD vs. Control Group			
Model	Genotype/Allele	OR (95% CI)	*p* Value	AIC	OR (95% CI) *	*p* Value	AIC
	**rs1570360**						
Codominant	GG	1			1		1045.7
	AG	1.12 (0.81–1.55)	0.499	989.3	1.25 (0.91–1.73)	0.171	
	AA	0.74 (0.49–1.13)	0.168		0.94 (0.63–1.41)	0.763	
Dominant	GG	1			1	0.38	1045.5
	AG + AA	0.98 (0.73–1.32)	0.9	990.7	1.14 (0.85–1.52)		
Recessive	GG + AG	1			1	0.4	1045.6
	AA	0.71 (0.48–1.05)	0.087	987.8	0.85 (0.58–1.24)		
Overdominant	GG + AA	1			1	0.11	1043.8
	AG	1.21 (0.89–1.64)	0.23	989.2	1.27 (0.94–1.72)		
Additive	A	0.91 (0.75–1.11)	0.34	989.8	1.02 (0.84–1.23)	0.88	1046.2
	**rs699947**						
Codominant	AA	1			1		
	AC	1.31 (0.92–1.86)	0.132	990.4	0.97 (0.69–1.37)	0.87	1045.9
	CC	1.16 (0.76–1.75)	0.492		1.28 (0.86–1.89)	0.226	
Dominant	AA	1			1		
	AC + CC	1.26 (0.90–1.75)	0.17	988.8	1.07 (0.78–1.47)	0.67	1046.1
Recessive	AA + AC	1			1		
	CC	0.97 (0.69–1.38)	0.88	990.7	1.30 (0.93–1.81)	0.13	1043.9
Overdominant	AA-CC	1			1		
	AC	1.23 (0.91–1.64)	0.18	988.9	0.87 (0.65–1.16)	0.34	1045.4
Additive	C	1.08 (0.88–1.33)	0.45	990.1	1.12 (0.92–1.37)	0.25	1044.9
	**rs3025033**						
Codominant	AA	1			1		
	AG	0.95 (0.69–1.30)	0.751	990.8	0.69 (0.51–0.95)	**0.025**	1040.7
	GG	0.61 (0.29–1.26)	0.181		0.50 (0.24–1.03)	0.061	
Dominant	AA	1			1		
	AG + GG	0.90 (0.67–1.22)	0.5	990.3	0.67 (0.49–0.90)	**0.0088**	1039.4
Recessive	AA + AG	1			1		
	GG	0.62 (0.30–1.27)	0.18	988.9	0.56 (0.27–1.15)	0.11	1043.7
Overdominant	AA + GG	1			1		
	AG	0.98 (0.72–1.34	0.91	990.7	0.73 (0.53–0.99)	**0.045**	1042.3
Additive	G	0.87 (0.68–1.13)	0.3	989.6	0.70 (0.54–0.90)	**0.0058**	1038.7
	**rs2146323**						
Codominant	CC	1			1		
	AC	1.11 (0.81–1.53)	0.509	989.9	0.84 (0.61–1.15)	0.28	1046.5
	AA	0.75 (0.48–1.20)	0.231		0.79 (0.51–1.21)	0.28	
Dominant	CC	1			1		
	AC + AA	1.01 (0.75–1.37	0.92	990.7	0.83 (0.62–1.11)	0.2	1044.6
Recessive	CC + AC	1			1		
	AA	0.71 (0.46–1.10)	0.12	988.3	0.86 (0.57–1.28)	0.45	1045.7
Overdominant	CC + AA	1			1		
	AC	1.19 (0.89–1.60)	0.24	989.4	0.89 (0.67–1.20)	0.45	1045.7
Additive	A	0.93 (0.75–1.15)	0.49	990.2	0.88 (0.72–1.07)	0.2	1044.7

OR—odds ratio; CI—confident interval; *p*—significance level and Bonferroni corrected significance level when *p* < 0.05/4; *p*-values marked with bold indicate statistically significant *p*-values; AIC—Akaike information criteria; * Ods adjusted for age in exudative AMD analysis.

**Table 4:** Linkage disequilibrium between the four *VEGFA* SNPs.

SNPs	Early AMD vs. Controls			Exudative AMD vs. Controls		
	D’	r^2^	*p* Value	D’	r^2^	*p* Value
rs1570360–rs699947	0.07182	0.2521	0.0	0.7087	0.2641	0.0
rs1570360–rs3025033	0.3771	0.0204	0.0	0.3919	0.0210	0.0
rs1570360–rs2146323	0.0898	0.0077	0.001	0.1191	0.0138	0.0
rs699947–rs3025033	0.1503	0.0066	0.0021	0.0389	0.0004	0.4298
rs699947–rs2146323	0.9904	0.5051	0.0	0.9871	0.4970	0.0
rs3025033–rs2146323	0.0866	0.0036	0.0232	0.1844	0.0150	0.0

SNP—single nucleotide polymorphism; D’ is the deviation between the expected haplotype frequency and the observed frequency (D’ scale: 0,1). r^2^ is the squared correlation coefficient of the haplotype frequencies (r^2^ scale: 0,1); *p*—significance level; significant when *p* < 0.05.

**Table 5:** Associations between *VEGFA* haplotypes and risk of early AMD.

Haplotype	SNP1	SNP2	SNP3	SNP4	Frequency (%)			OR (95% CI)	*p* Value
					Early AMD	Controls	Total		
1	G	C	A	C	31.13	31.71	31.43	1.00	-
2	G	A	A	A	18.87	15.22	16.98	1.30 (0.92–1.84)	0.14
3	A	A	A	C	16.08	15.55	15.8	1.08 (0.78–1.50)	0.64
4	G	C	G	C	13.14	10.35	11.68	1.31 (0.87–1.97)	0.19
5	A	A	A	A	9.17	10.01	9.59	0.96 (0.63–1.46)	0.86
6	G	A	G	A	3.53	6.14	4.88	0.61 (0.33–1.13)	0.12
7	A	C	A	C	4.7	4.56	4.63	1.14 (0.64–2.01)	0.66
8	A	A	G	A	3.39	5.09	4.29	0.63 (0.34–1.17)	0.14
rare	*	*	*	*	NA	NA	0.72	0.00 (-)	1

OR—odds ratio; CI—confident interval; *p*—significance level; significant when *p* < 0.05; *p*-values marked with bold indicate statistically significant *p*-values; AIC—Akaike information criteria; SNP1 rs1570360; SNP2 rs699947; SNP3 rs3025033; SNP4 rs2146323; rare—pooled haplotypes with frequencies < 1 %. *—allele of rare haplotype. Global haplotype association *p* value: 0.025.

**Table 6:** Associations between *VEGFA* haplotypes and risk of exudative AMD.

Haplotype	SNP1	SNP2	SNP3	SNP4	Frequency (%)			OR (95% CI)	*p* Value
					Exudative AMD	Controls	Total		
1	G	C	A	C	35.1	31.71	33.52	1.00	-
2	A	A	A	C	16.2	15.55	15.89	0.93 (0.68–1.27)	0.65
3	G	A	A	A	14.65	15.22	14.88	0.86 (0.61–1.22)	0.4
4	A	A	A	A	11.4	10.01	10.76	1.07 (0.73–1.57)	0.73
5	G	C	G	C	8.62	10.35	9.38	0.81 (0.53–1.23)	0.32
6	G	A	G	A	5.08	6.14	5.58	0.69 (0.40–1.20)	0.19
7	A	C	A	C	5.44	4.56	5.04	1.17 (0.69–1.98)	0.56
8	A	A	G	A	2.99	5.09	3.98	0.46 (0.23–0.90)	**0.023**
rare	*	*	*	*	NA	NA	0.95	0.36 (0.09–1.46)	0.15

OR—odds ratio; CI—confident interval; *p*—significance level; significant when *p* < 0.05; *p*-values marked with bold indicate statistically significant *p*-values; AIC—Akaike information criteria; SNP1 rs1570360; SNP2 rs699947; SNP3 rs3025033; SNP4 rs2146323; rare—haplotypes with frequencies < 1 %;. *—allele of rare haplotype. Global haplotype association *p* value: 0.13.

**Table 7:** VEGF-A and VEGF-R2/KDR concentration associations with *VEGFA* polymorphisms in exudative AMD and controls.

Model	VEGF-A				VEGF-R2/KDR			
	Exudative AMD (pg/mL), Median (IQR)	*p* Value	Control Group (pg/mL), Median (IQR)	*p* Value	Exudative AMD (pg/mL), Median (IQR)	*p* Value	Control Group (pg/mL), Median (IQR)	*p* Value
**rs1570360**								
Dominant AG + AA vs. GG	383.55 (427.3) vs. 640.36 (1234.32)	0.142	711.25 (472.66) vs. 526.14 (391.91)	0.181	12,588.6 (6294.67) vs. 13,402.08 (5875.31)	0.569	14,435.95 (7035.44) vs. 16,211.6 (7547.35)	0.916
Recessive AA vs. AG + GG	726.99 (-) vs. 421.42 (646.25)	0.737	727.89 (-) vs. 581.23 (518.70)	0.615	17,876.6 (-) vs. 12,673.9 (4531.86)	0.560	15,428.35 (-) vs. 15,473.4 (7038.14)	0.451
**rs699947**								
Dominant AC + CC vs. AA	485.95 (945.93) vs. 194.97 (-)	**0.046**	546.82 (553.1) vs. 711.25 (272.64)	0.531	13,357.85 (5451.18) vs. 11,403.18 (-)	0.130	14,735.2 (7207.88) vs. 16,872.8 (10,171.29)	0.347
Recessive CC vs. AC + AA	422.67 (1231.86) vs. 434.75 (678.21)	0.447	526.14 (9396.71) vs. 711.254 (479.99)	0.156	14,044.95 (7679.2) vs. 12,418.33 (4638.02)	0.231	16,211.6 (6731.85) vs. 14,435.95 (7038.14)	0.654
**rs3025033**								
Dominant AG + GG vs. AA	401.86 (734.59) vs. 517.55 (1022.04)	0.935	629.32 (336.84) vs. 526.14 (621.82)	0.944	12,588.6 (1324.06) vs. 17,349.15 (7709.45)	0.623	14,089.38 (6374.95) vs. 17,524.8 (8218.45)	0.307
Recessive GG vs. AG + AA	- (-) vs. 454.31 (819.36)	-	- (-) vs. 581.23 (377.25)	-	- (-) vs. 12,673.9 (5468.76)	-	- (-) vs. 15,819.96 (6356.65)	-
**rs2146323**								
Dominant AC + AA vs. CC	383.55 (1282.99) vs. 454.311 (527.24)	0.866	735.12 (743.12) vs. 536.48 (318.29)	0.227	13,357.85 (5271.05) vs. 12,673.9 (5624.84)	0.874	13,443.55 (7708) vs. 15,819.98 (7312.38)	0.270
Recessive AA vs. AC + CC	- (-) vs. 42.67 (677.02)	-	739.89 (-) vs. 546.82 (484.33)	0.402	- (-) vs. 12,759.2 (5358.85)	-	15,880.4 (-) vs. 15,428.35 (6868.4)	0.952

IQR—interquartile range; any missing IQR value is due to the small sample size in the study subgroup; *p*—significance level; significant when *p* < 0.05; *p*-values marked with bold indicate statistically significant *p*-values.

## Data Availability

All data relevant to the study are included in the article or uploaded as supplementary information.

## Supplementary Materials

The following are available online at https://www.mdpi.com/article/10.3390/cells11060996/s1, Table S1: Frequencies of *VEGFA* (rs1570360, rs699947, rs3025033 and rs2146323) genotypes and alleles in females with early AMD and exudative AMD, and controls, Table S2: Binomial logistic regression analysis of *VEGFA* (rs1570360, rs699947, rs3025033, and rs2146323) in females with early and exudative AMD and controls, Table S3: Frequencies of *VEGFA* (rs1570360, rs699947, rs3025033, and rs2146323) genotypes and alleles in males with early AMD and exudative AMD, and controls, Table S4: Binomial logistic regression analysis of *VEGFA* (rs1570360, rs699947, rs3025033, and rs2146323) in males with early and exudative AMD and controls.
