# Supplementary material for: VEGFA Haplotype and VEGF-A and VEGF-R2 Protein Associations with Exudative Age-Related Macular Degeneration
Source: Cells. 2022 Mar 15;11(6):996. doi: 10.3390/cells11060996 (PMC8947491; doi:10.3390/cells11060996)
Supplement: Supplementary file 1 [file cells-11-00996-s001.zip › cells-1579952-supplementary.pdf]

Supplementary material 1.

Table S1. Frequencies of *VEGFA* (rs1570360, rs699947, rs3025033 and rs2146323) genotypes and alleles in females with early AMD and exudative AMD, and controls

SNP Genotype/allele	*Early AMD N=235 n (%)*	*Exudative AMD N=270 n (%)*	*Control group N=235 n (%)*	*p value**	*p value***	*p value****
rs1570360						
GG	115 (48.9)	119 (44.1)	108 (46)	0.142	0.285	0.498
AG	88 (37.4)	107 (39.6)	79 (33.6)			
AA	32 (13.6)	44 (16.3)	48 (20.4)			
G	318 (67.7)	345 (63.9)	295 (62.8)	0.097	0.646	0.208
A	152 (32.3)	195 (36.1)	177 (37.2)			
rs699947						
AA	61 (26)	81 (30)	70 (29.8)	0.618	0.490	0.182
AC	122 (51.9)	118 (43.7)	113 (48.1)			
CC	52 (22.1)	71 (26.3)	52 (22.1)			
A	244 (51.9)	280 (51.8)	253 (53.8)	0.556	0.530	0.984
C	226 (48.1)	260 (48.1)	217 (46.2)			
rs3025033						
AA	148 (63)	199 (73.3)	147 (62.6)	0.374	**0.017**	**0.032**
AG	80 (34)	64 (23.7)	75 (31.9)			
GG	7 (3)	7 (2.6)	13 (5.5)			
A	376 (80)	462 (85.6)	369 (78.5)	0.573	**0.003**	**0.019**
G	94 (20)	78 (14.4)	101 (21.5)			
rs2146323						
CC	96 (40.9)	124 (45.9)	92 (39.1)	0.560	0.308	0.299
AC	111 (47.2)	109 (40.4)	107 (45.5)			
AA	28 (11.9)	37 (13.7)	36 (15.3)			
C	303 (64.5)	357 (66.1)	291 (61.9)	0.417	0.165	0.584
A	167 (35.5)	183 (33.9)	179 (38.1)			

*p – significance level and Bonferroni corrected significance level when p<0.05/4; *Early AMD vs. Control group;*

***Exudative AMD vs. Control group;*

****Early AMD vs. Exudative AMD.*

Table S2. Binomial logistic regression analysis of *VEGFA* (rs1570360, rs699947, rs3025033, and rs2146323) in females with early and exudative AMD and controls

				rs1570360			
		Early AMD vs. Control group			***Exudative AMD vs. Control group***		
Model	***Genotype/allele***	***OR (95% CI)***	***p value***	***AIC***	***OR (95% CI)****	***p value***	***AIC***
				**rs1570360**			
Codominant	G/G	1.00			1.00		
	A/G	1.05 (0.70-1.56)	0.826	653.6	1.10 (0.73-1.67)	0.648	647.1
	A/A	0.63 (0.37-1.05)	0.077		0.89 (0.53-1.48)	0.640	
Dominant	G/G	1.00			1.00	0.91	645.8
	A/G-A/A	0.89 (0.62-1.27)	0.52	655.1	1.02 (0.70-1.48)		
Recessive	G/G-A/G	1.00			1.00	0.5	645.3
	A/A	0.61 (0.38-1.00)	**0.049**	651.7	0.85 (0.53-1.37)		
Overdominant	G/G-A/A	1.00			1.00	0.5	645.4
	A/G	1.18 (0.81-1.73)	0.39	654.8	1.14 (0.78-1.68)		
Additive	A	0.84 (0.66-1.07)	0.15	653.5	0.97 (0.76-1.24)	0.79	645.7
				**rs699947**			
Codominant	A/A	1.00			1.00		
	A/C	1.24 (0.81-1.90)	0.327	656.6	0.82 (0.53-1.28)	0.387	643.6
	C/C	1.15 (0.69-1.92)	0.601		1.35 (0.81-2.24)	0.256	
Dominant	A/A	1.00			1.00		
	A/C-C/C	1.21 (0.81-1.81)	0.35	654.7	0.98 (0.65-1.46)	0.91	645.8
Recessive	A/A-A/C	1.00			1.00		
	C/C	1.00 (0.65-1.55)	1	655.6	1.51 (0.97-2.34)	0.064	642.4
Overdominant	A/A-C/C	1.00			1.00		
	A/C	1.17 (0.81-1.67)	0.41	654.9	0.72 (0.50-1.05)	0.09	642.9
Additive	C	1.08 (0.84-1.40)	0.56	655.2	1.14 (0.88-1.46)	0.32	644.8
				**rs3025033**			
Codominant	A/A	1.00			1.00		

	A/G	1.06 (0.72-1.56)	0.771	655.6	0.57 (0.37-0.87)	**0.009**	638.7
	G/G	0.53 (0.21-1.38)	0.195		0.42 (0.15-1.12)	0.083	
Dominant	A/A	1.00			1.00		
	A/G-G/G	0.98 (0.68-1.43)	0.92	655.5	0.55 (0.37-0.82)	**0.0032**	637.1
Recessive	A/A-A/G	1.00			1.00		
	G/G	0.52 (0.21-1.34)	0.17	653.7	0.49 (0.18-1.31)	0.15	643.7
Overdominant	A/A-G/G	1.00			1.00		
	A/G	1.10 (0.75-1.62)	0.62	655.3	0.60 (0.39-0.91)	**0.015**	639.9
Additive	G	0.91 (0.67-1.25)	0.57	655.2	0.60 (0.42-0.84)	**0.0028**	636.9
				**rs2146323**			
Codominant	C/C	1.00			1.00		
	A/C	0.99 (0.67-1.47)	0.977	656.4	0.65 (0.44-0.98)	**0.040**	642.9
	A/A	0.75 (0.42-1.32)	0.313		0.65 (0.37-1.14)	0.135	
Dominant	C/C	1.00			1.00		
	A/C-A/A	0.93 (0.64-1.35)	0.71	655.4	0.65 (0.45-0.95)	**0.026**	640.9
Recessive	C/C-A/C	1.00			1.00		
	A/A	0.75 (0.44-1.27)	0.28	654.4	0.80 (0.47-1.36)	0.42	645.1
Overdominant	C/C-A/A	1.00			1.00		
	A/C	1.07 (0.75-1.54)	0.71	655.4	0.73 (0.50-1.06)	0.1	643.1
Additive	A	0.90 (0.69-1.17)	0.42	654.9	0.77 (0.59-1.00)	0.049	641.9

*OR – odds ratio; CI – confident interval; p – significance level and Bonferroni corrected significance level when p<0.05/4; AIC – Akaike information criteria; *ORs adjusted for age in exudative AMD analysis.*

Table S3. Frequencies of *VEGFA* (rs1570360, rs699947, rs3025033, and rs2146323) genotypes and alleles in males with early AMD and exudative AMD, and controls

SNP Genotype/allele	*Early AMD N=104 n (%)*	*Exudative AMD N=149 n (%)*	*Control group N=139 n (%)*	*p value**	*p value***	*p value****
rs1570360						
GG	47 (45.2)	64 (43)	69 (49.6)	0.684	0.378	0.920
AG	40 (38.5)	61 (40.9)	46 (33.1)			
AA	17 (16.3)	24 (16.1)	24 (17.3)			
G	134 (64.4)	189 (63.4)	184 (66.2)	0.686	0.488	0.818
A	74 (35.6)	109 (36.6)	94 (33.8)			
rs699947						
AA	25 (24)	38 (25.5)	42 (30.2)	0.488	0.671	0.844
AC	52 (50)	69 (46.3)	60 (43.2)			
CC	27 (26)	42 (28.2)	37 (26.6)			
A	102 (49)	145 (48.7)	144 (51.8)	0.512	0.451	0.933
C	106 (51)	153 (51.3)	134 (48.2)			
rs3025033						
AA	67 (64.4)	91 (61.1)	81 (58.3)	0.623	0.756	0.775
AG	32 (30.8)	52 (34.9)	50 (36)			
GG	5 (4.8)	6 (4)	8 (5.8)			
A	166 (79.8)	234 (78.5)	212 (76.3)	0.352	0.516	0.727
G	42 (20.2)	64 (21.5)	66 (23.7)			
rs2146323						
CC	46 (44.2)	67 (45)	66 (47.5)	0.333	0.744	0.701
AC	46 (44.2)	60 (40.3)	50 (36)			
AA	12 (11.5)	22 (14.8)	23 (16.5)			
C	138 (66.3)	194 (65.1)	182 (65.5)	0.840	0.926	0.831
A	70 (33.7)	104 (34.9)	96 (34.5)			

p – significance level and Bonferroni corrected significance level when $p<0.05/4$;

**Early AMD vs. Control group;*

***Exudative AMD vs. Control group;*

****Early AMD vs. Exudative AMD.*

Table S4. Binomial logistic regression analysis of *VEGFA* (rs1570360, rs699947, rs3025033, and rs2146323) in males with early and exudative AMD and controls

rs1570360							
		Early AMD vs. Control group			***Exudative AMD vs. Control group***		
Model	***Genotype/allele***	***OR (95% CI)***	***p value***	***AIC***	***OR (95% CI)****	***p value***	***AIC***
				**rs1570360**			
	G/G	1.00			1.00		
Codominant	A/G	1.28 (0.73-2.24)	0.395	337.1	1.49 (0.89-2.49)	0.132	400.3
	A/A	1.04 (0.50-2.14)	0.916		1.08 (0.56-2.10)	0.815	
Dominant	G/G	1.00			1.00	0.21	399.1
	A/G-A/A	1.20 (0.72-1.99)	0.49	335.3	1.35 (0.84-2.15)		
Recessive	G/G-A/G	1.00			1.00	0.76	400.6
	A/A	0.94 (0.47-1.85)	0.85	335.8	0.91 (0.49-1.69)		
Overdominant	G/G-A/A	1.00			1.00	0.13	398.3
	A/G	1.26 (0.74-2.15)	0.39	335.1	1.46 (0.90-2.36)		
Additive	A	1.07 (0.76-1.50)	0.71	335.7	1.12 (0.81-1.54)	0.49	400.2
			**rs699947**				
Codominant	A/A	1.00			1.00		
	A/C	1.46 (0.78-2.70)	0.234	336.	1.25 (0.72-2.20)	0.428	402
	C/C	1.23 (0.61-2.47)	0.569		1.24 (0.66-2.32)	0.505	
Dominant	A/A	1.00			1.00		
	A/C-C/C	1.37 (0.77-2.44)	0.28	334.7	1.25 (0.74-2.10)	0.4	400
Recessive	A/A-A/C	1.00			1.00		
	C/C	0.97 (0.54-1.72)	0.91	335.8	1.08 (0.64-1.82)	0.78	400.6
Overdominant	A/A-C/C	1.00			1.00		
	A/C	1.32 (0.79-2.19)	0.29	334.7	1.13 (0.71-1.80)	0.61	400.4

Additive	C	1.11 (0.78-1.57)	0.56	335.5	1.11 (0.81-1.52)	0.5	400.2
**rs3025033**							
Codominant	A/A	1.00			1.00		
	A/G	0.77 (0.45-1.34)	0.360	336.9	0.90 (0.55-1.48)	0.690	402
	G/G	0.76 (0.24-2.42)	0.637		0.67 (0.22-2.00)	0.468	
Dominant	A/A	1.00			1.00		
	A/G-G/G	0.77 (0.46-1.30)	0.33	334.9	0.87 (0.54-1.40)	0.57	400.3
Recessive	A/A-A/G	1.00			1.00		
	G/G	0.83 (0.26-2.61)	0.74	335.7	0.69 (0.23-2.05)	0.5	400.2
Overdominant	A/A-G/G	1.00			1.00		
	A/G	0.79 (0.46-1.36)	0.4	335.1	0.93 (0.57-1.52)	0.78	400.6
Additive	G	0.82 (0.53-1.26)	0.36	335	0.86 (0.58-1.28)	0.47	400.1
**rs2146323**							
Codominant	C/C	1.00			1.00		
	A/C	1.32 (0.76-2.29)	0.322	335.6	1.19 (0.72-1.98)	0.502	402.1
	A/A	0.75 (0.34-1.65)	0.474		0.98 (0.50-1.93)	0.949	
Dominant	C/C	1.00			1.00		
	A/C-A/A	1.14 (0.68-1.90)	0.61	335.6	1.12 (0.70-1.79)	0.62	400.4
Recessive	C/C-A/C	1.00			1.00		
	A/A	0.66 (0.31-1.39)	0.27	334.6	0.90 (0.48-1.71)	0.76	400.6
Overdominant	C/C-A/A	1.00			1.00		
	A/C	1.41 (0.84-2.37)	0.19	334.1	1.20 (0.74-1.93)	0.46	400.1
Additive	A	0.97 (0.68-1.38)	0.85	335.8	1.03 (0.75-1.42)	0.86	400.6

*OR – odds ratio; CI – confident interval; p – significance level and Bonferroni corrected significance level when p<0.05/4; AIC – Akaike information criteria; *ORs adjusted for age in exudative AMD analysis.*
